# Bioactive Activities of the Phenolic Extract from Sterile Bracts of *Araucaria angustifolia*

**DOI:** 10.3390/antiox11122431

**Published:** 2022-12-09

**Authors:** Thaís Estéfane Fischer, Amanda Marcondes, Danianni Marinho Zardo, Alessandro Nogueira, Ricardo C. Calhelha, Josiana A. Vaz, Lillian Barros, Acácio Antonio Ferreira Zielinski, Aline Alberti

**Affiliations:** 1 Graduate Program in Food Science and Technology, State University of Ponta Grossa (UEPG), Ponta Grossa 84030-900, PR, Brazil; 2 Centro de Investigação de Montanha (CIMO), Instituto Politécnico de Bragança, Campus de Santa Apolónia, 5300-253 Bragança, Portugal; 3 Laboratório Associado para a Sustentabilidade e Tecnologia em Regiões de Montanha (SusTEC), Instituto Politécnico de Bragança, Campus de Santa Apolónia, 5300-253 Bragança, Portugal; 4 Department of Chemical Engineering and Food Engineering, Federal University of Santa Catarina (UFSC), Florianópolis 88010-970, SC, Brazil

**Keywords:** Paraná pine, response surface methodology, phenolic compounds, antioxidant activity, antimicrobial activity, anti-inflammatory activity, anti-proliferative activity

## Abstract

Sterile bracts can represent 80% of *Araucaria angustifolia* pinecone and are a rich source of phenolic compounds. This study aimed to optimize the extraction of the phenolic compounds from *Araucaria angustifolia* bracts using response surface methodology; the bioactivity properties were also investigated. The effects of the ethanol concentration, solute/solvent ratio, and temperature in relation to the phenolic composition and antioxidant activity were evaluated. The quantification and identification of the individual phenolic compounds (using high-performance liquid chromatography) and their bioactivity were evaluated. The optimized extraction conditions, which detected gallic acid, catechin, epicatechin, quercetin, and kaempferol, were obtained using 60% ethanol at a ratio of 1:38 (*w*/*v*) and a temperature of 80 °C. The extract showed high levels of phenolic classes and antioxidant activity. The extract also showed an inhibitory activity for pathogenic (approximately 80%, 10,000 µg/mL) and lactic acid (27.9%, 15,000 µg/mL) bacteria strains. The α-glucosidase inhibitory activity was approximately ten times greater than acarbose, demonstrating its high antiglycemic potential. No antioxidant and anti-inflammatory cellular activity were determined; however, a high cytotoxicity for non-tumor cells and the antiproliferative activity against the tumor cells were observed. Overall, the phenolic extract showed promising action in relation to the fight against the diseases related to oxidative stress and, hopefully, the application of the safe concentrations of the extract, based on bioavailability assays, can be verified.

## 1. Introduction

**Figure 1:**
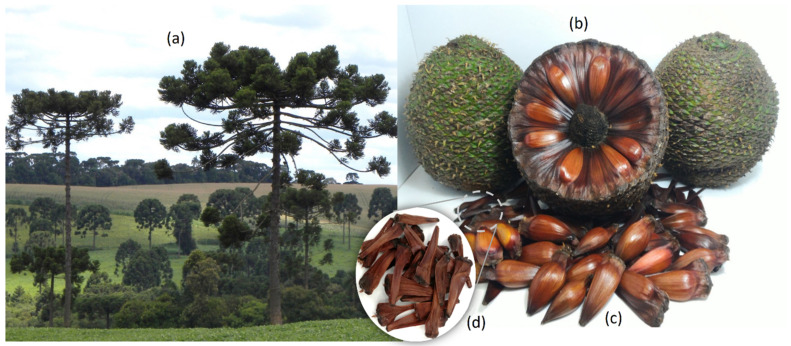
*Araucaria angustifolia* trees (**a**); mature pinecone (**b**) with seeds (**c**) and bracts (sterile seeds) (**d**).

*Araucaria angustifolia* (Bertol.) Kuntze tree is a conifer found in mixed ombrophilous forest, mainly in the southern region of Brazil. The tree can reach 35 m in height, with a diameter of 1.2 m, and is easily recognizable due to its large umbelliferous shape (Figure 1a). The reproductive structure of *Araucaria* is composed of male and female strobiles, which are constituted of the seeds and bracts (sterile seeds) that compose the pinecone (Figure 1b) [1,2]. The seeds, known as “*pinhão*” (Figure 1c), are rich in starch and amino acids, and are consumed after either being boiled or baked [3]. The sterile bracts (Figure 1d) correspond to around 80% of the pinecone and remain on the ground when the mature pinecones fall or are removed from the trees by animals [4]. The bracts are not used for any industrial purpose, however, while they are in the pinecone, they form the pinecone and protect the pine seeds [5].

Sterile bracts are rich in flavonoids. The main phenolic subclasses are flavan-3-ols (catechin, epicatechin, and apigenin) and flavonols (quercetin glycosides). The phenolic extracts of sterile bracts of *Araucaria angustifolia* have a high antioxidant, antigenotoxic [4,6], and antimicrobial activity, mainly due to the presence of flavan-3-ols (catechin) and flavonols (quercetin) [7]. The bark of *Araucaria angustifolia* pine nuts has been linked to the antiglycemic activity due to their capacity to inhibit human salivary and porcine pancreatic α-amylases; it was also effective in reducing the blood glucose levels in rats after the administration of starch, which was possibly due to the tannin fraction of the extract [8]. Plant materials rich in phenolic compounds, mainly from the flavonoid class, demonstrate bioactive properties. However, in addition to evaluating the bioactivity, the cytotoxicity of optimized phenolic plant extracts must be evaluated, especially the parts of the plants that are not commonly consumed such as the sterile bracts of *Araucaria angustifolia*.

The extraction of the bioactive compounds can be influenced by factors such as the method used, the chemical nature and the particle size of the sample, the solvent, the extraction time and temperature, the pH, and the solute/solvent ratio. Furthermore, the storage conditions, and the presence of compounds such as pigments, terpenes, and fats, can interfere with the final phenolic content. To optimize the extraction process, the parameters mentioned above are studied to achieve better yields during the process of the extraction of the phenolic compounds [9]. The most common solvents used to extract these compounds are methanol, acetone, and their water mixtures. However, these products are toxic and are not totally eliminated during the solvent removal step. Therefore, when the objective is to apply the extract in food products, the selected solvent must be food grade [10,11]. Thus, the aim of this study was to use ethanol solutions to extract the phenolic compounds from the sterile bracts of *Araucaria angustifolia* using response surface methodology, and also to verify the antioxidant, antimicrobial, cytotoxicity, and bioactive activities.

## 2. Materials and Methods

### 2.1. Materials

The mature pinecones of *Araucaria angustifolia* were collected in the Campos Gerais region of the state of Paraná, Brazil. This project was registered in the National System of Management of Genetic Heritage (SisGen) of the Brazilian Ministry of Environment—www.sisgen.gov.br, accessed on 6 November 2022 (register# A20820A). The pinecones were threshed to separate the seeds (*pinhões*) and bracts (sterile seeds) (Figure 1).

### 2.2. Extraction of Phenolic Compounds

The bracts were selected and dried at 60 °C, which was followed by comminution and sieving (40–60 mesh). To optimize the phenolic extraction from the bracts a Box–Behnken [12] design was applied. The effect of the ethanol concentration, *x*_1_, (20, 40, and 60%), solid-to-solvent ratio, *x*_2_, (1:10, 1:25, and 1:40, *w*/*v*), and the temperature, *x*_3_, (30, 55, and 80 °C) were evaluated (Table 1). The extraction experiments were performed with 2 g of bract powder for 20 min. The extracts were subsequently centrifuged (8160× *g*, 20 min at 4 °C) (HIMAC CR-GII, Hitachi, Ibaraki, Japan) and stored at −18 °C before the analysis.

### 2.3. Phenolic Analysis

The phenolic composition (total phenolic compounds, flavonoids, and tannins) of the fifteen extracts were analyzed. The total phenolic compounds were quantified according to the Folin–Ciocalteu method [13]. The sample absorbances were compared with the calibration curve (TPC = 0.0031 × absorbance + 0.0066; R^2^ = 0.9956) of gallic acid (10–100 mg/L). The results were expressed as the mg of the gallic acid equivalent per g of the bracts (mg GAE/g).

The total flavonoid content was determined using an aluminum chloride colorimetric method [14]. A calibration curve (TF = 0.0023 × absorbance + 0.0006; R^2^ = 0.9991) of catechin (20–200 mg/L) was used as the standard and the results were expressed as the mg of the catechin equivalent per g of the bracts (mg CTE/g).

The condensed tannin content was assessed using the acidified vanillin method [15]. The sample absorbances were compared with the calibration curve (TAN = 0.00192 × absorbance + 0.01558; R^2^ = 0.9929) of catechin (20–200 mg/L). The results were expressed in the catechin equivalents per g of the bracts (mg CTE/g).

The gallic acid, catechin, epicatechin, quercetin, and kaempferol content were analyzed at the optimum condition (after extraction optimization) using HPLC. The chromatographic system was 2695 Alliance (Waters, Milford, MA, USA) equipment, composed of a quaternary pump, autosampler, and photodiode array detector (PDA 2998, Waters, USA). The mobile phase was composed of 1% acetic acid (A) and acetonitrile (B), according to the following elution gradient: 3–15% B (0–30 min), 15–40% B (30–40 min), and 40–75% B (40–45 min), followed by an isocratic elution at 85% B (5 min) and the reconditioning of the column (10 min). The flow rate was 1.0 mL/min. The separation occurred in a Gemini C18 (4.6 × 150 mm, 5 µm, Phenomenex, Torrance, CA, USA) column at 20 °C. The identification and quantification were performed by comparing the retention times and the spectra of the standards.

### 2.4. Antioxidant Activity

The antioxidant activity of the samples was measured by DPPH [16] and ABTS [17]. The absorbances were compared with the standard curves (DPPH = 2.77 × absorbance; R^2^ = 0.9934; ABTS = 4.30 × absorbance; R^2^ = 0.9974) of Trolox (50–500 µmol/L). All the results were expressed as the µmol Trolox equivalent per g of the bracts (µmol TE/g).

### 2.5. Antimicrobial Activity

The minimum inhibitory concentration was obtained using the broth microdilution technique, according to the guidelines of the Clinical and Laboratory Standards Institute [18] and calculated as the percentage of the bacterial inhibition. The strains of *Escherichia coli* (ATCC 25922), *Staphylococcus aureus* (ATCC 25923), *Salmonella enterica* spp. *enterica* (ATCC 13076), and *Lactobacillus brevis* were inoculated on the nutrient agar and incubated in a stove (QUIMIS—Q-316.24, São Paulo, Brazil) at 35 ± 1 °C for 24 h and 48 h for the pathogenic strains and lactic acid bacteria, respectively. Subsequently, the strains were suspended in a 0.9% sodium chloride saline solution at a concentration of 10^8^ CFU/mL (0.5 McFarland’s scale) and diluted to obtain the working culture. The extract was dried in a stove (MARCONI—MA—035/5) and later diluted in Mueller–Hinton broth (10,000 to 312.5 µg/mL). In a microplate, 200 µL of the diluted sample and 10 µL of the microorganism were added at 10^6^–10^5^ CFU/mL. A positive and negative control (0.1% chlorhexidine) were also prepared. The microplates were incubated at 35 ± 1 °C from 18 to 24 h. After the incubation period, the absorbance readings were performed at 600 nm using a microplate reader (Epoch Microplate Spectrophotometer, Synergy-BIOTEK, Winooski, VT, USA).

### 2.6. Antiglycemic Activity

The antiglycemic activity was evaluated for the α-glucosidase inhibitory activity [19]. The phenolic extract was lyophilized (Terroni, LS 3000, São Paulo, Brazil) and later resuspended and diluted in 5% DMSO to obtain serial dilutions (3125 to 97.65625 µg/mL). In a microplate, 10 µL of the phenolic extracts, 20 µL of the α-glucosidase enzyme solution (0.5 U/mL), and 120 µL of the phosphate buffer (0.1 M, pH 6.9) were added and then incubated (BioTek Absorbance Microplate Reader—Elx808, Agilent, Santa Clara, CA, USA) at 37 °C for 15 min. After the incubation period, 80 µL of Na_2_CO_3_ (0.2 M) was added to finish the reaction, and the absorbance readings were performed at 405 nm in a microplate reader (Epoch Microplate Spectrophotometer, Synergy-BIOTEK, Winooski, VT, USA). The antiglycemic activity was determined by plotting the percentage inhibition of the enzyme (Equation (1)) versus the extract concentration (µg/mL), which was expressed as IC_50_. A control (without phenolic extract), a blank (without the α-glucosidase enzyme), and an acarbose control were performed.

% Inhibition = [(Control Absorbance − Sample Absorbance)/(Control Absorbance)] × 100(1)

### 2.7. Cellular Antioxidant Activity

To assess the cellular antioxidant activity (CAA) [20], the extracts were dissolved in water to obtain a concentration of 8 mg/mL, from which successive dilutions were made with 2’,7’-dichlorohydrofluorescein (DCFH), which was prepared with ethanol and diluted with HBSS (50 μM), obtaining the concentrations to be tested (500–2000 μg/mL).

The cell line used was RAW 246.7 (murine macrophage cells), which was maintained in an incubator at 37 °C, with a humidified atmosphere and 5% CO_2_ and a DMEM culture medium supplemented with L-glutamine, penicillin (100 U/mL), streptomycin (100 μg/mL), fetal serum bovine (10%), and non-essential amino acids (2 mM).

The murine macrophages were detached with a cell scraper and the content was transferred to a falcon tube. The solution was centrifuged for 5 min at 1200 rpm. The medium was discarded and the amount of new medium was added according to the size of the obtained pellet. A solution with a cell density of 70,000 cells/mL was then prepared. An aliquot of the prepared solution (300 μL) was transferred to black microplates with clear-bottoms (SPL Life Sciences, Pocheon, Gyeonggi, Korea) and incubated for 48 h. After the incubation period, the medium was discarded and the cells were washed with HBSS (2×, 100 μL), treated with different extract concentrations (200 μL; 500–2000 μg/mL), and incubated for 1 h. The cells were subsequently washed with HBSS (3×, 100 μL), and a 2.2′-azobis(2-methylpropionamide) dihydrochloride (AAPH) solution (100 μL; 600 μM) was added. The fluorescence was read every five min for 1 h (Biotek FLx800 microplate reader, Agilent, Santa Clara, CA, USA) at 485 nm excitation and 538 nm emission. Quercetin was used as a positive control, and dichlorohydroflurescein and a DMEM culture medium were used as a negative control.

### 2.8. Anti-Inflammatory Activity

The anti-inflammatory activity was evaluated through the production of nitric oxide (NO), induced by LPS (lipopolysaccharide) by RAW 264.7 mouse macrophages [21]. The extracts were dissolved in water to obtain a concentration of 8 mg/mL, from which other dilutions were prepared (6.25; 25; 100; and 400 µg/mL). The RAW 264.7 cell line was cultured in a DMEM medium supplemented with 10% heat-inactivated fetal serum bovine and glutamine, which was maintained at 37 °C under 5% CO_2_ and humidified air. The cells were detached with a cell scraper and seeded into 96-well microplates at 150,000 cells per well and allowed to attach to the plate overnight. The cells were then treated with different concentrations of the extract for one hour. Dexamethasone (50 μM) was used as a positive control. The next step was stimulation with LPS (1 μg/mL) for 18 h. The extract and LPS were dissolved in the supplemented DMEM. A negative control without the addition of LPS was prepared and used to verify its possible effect on the basal levels of NO. The nitrite produced was determined by measuring the optical density at 515 nm and the results were expressed as an IC_50_ value (μg/mL), which corresponds to the concentration that induces a 50% inhibition of the NO production.

### 2.9. Antiproliferative Activity and Hepatotoxicity

The evaluation of the cytotoxic activity was performed using the sulforhodamine B assay (SRB) [21]. Human tumor cell lines from the carcinoma of the stomach (AGS), intestine (CaCo-2), and breast (MCF-7), as well as non-tumor cells (VERO and PLP2), were used. The hepatotoxicity was measured using a primary culture of non-tumor liver cells from a porcine liver (PLP2). The liver tissues were washed in Hank’s balanced saline solution containing 100 U/mL of penicillin and 100 μg/mL of streptomycin, which was divided into 1 × 1 mm^3^ explants. Some of these explants were placed in 25 cm^3^ tissue culture flasks in Dulbecco’s Modified Eagle Medium (DMEM) supplemented with 10% fetal serum bovine, 2 mM of non-essential amino acids, 100 U/mL of penicillin, and 100 μg/mL of streptomycin, which was then incubated at 37 °C in a humidified atmosphere containing 5% of carbon dioxide gas. Before confluence, the cells were subcultured and plated in 96-well microplates at a density of 1.0 × 10^4^ cells per well and cultured in a DMEM with 10% fetal serum bovine, 100 U/mL of penicillin, and 100 μg/mL of streptomycin. The cells were treated for 48 h with the different diluted sample solutions. After the exposure period, the cells were fixed by adding 50% cold trichloroacetic acid (*w*/*v*) (TCA, 25 μL) and incubated for 60 min at 4 °C. The plates were then washed with deionized water and dried. The SRB solution (0.1% *w*/*v* in 1% acetic acid, 50 μL) was then added to each well of the plate and incubated for 30 min. Unbound SRB was removed by washing with 1% acetic acid. The plates were air dried and the bound stain was solubilized with 100 µL of a 100 mM Tris base solution. Ellipticine was used as a positive control. The optical densities were read on a microplate reader at a wavelength of 540 nm. The results were expressed as a GI_50_ value (μg/mL), which corresponds to the concentration of the extract that inhibited 50% of cell proliferation.

The cytotoxic selectivity index (CSI) was calculated by the ratio between the GI_50_ for normal cell lines and the GI_50_ for tumoral cell lines. CSI values greater than 10 were considered selective [22].

### 2.10. Statistical Analysis

The data were presented as the mean and standard deviation (SD). A one-way ANOVA was performed to detect significant differences (*p* < 0.05) between the samples. Pearson’s correlation method (r) was used to analyze the correlation between the analyzed parameters, considering a significant *p*-value to be below 0.05.

Response surface methodology coupled with multiple regression analysis was used to find the best conditions to extract the bract phenolics from *Araucaria angustifolia*. A second-order polynomial equation (Equation (2)) was used to fit the experimental data.

(2) $$Y=\beta_{0}+\overset{3}{\underset{i=1}{\sum }}\beta_{i}X_{i}+\overset{3}{\underset{i=1}{\sum }}\beta_{ii}X_{i}^{2}+\overset{2}{\underset{i=1}{\sum }}\overset{3}{\underset{j=i+1}{\sum }}\beta_{ij}X_{i}X_{j}$$

where *Y* is the predicted response, *β*_0_, *β_i_*, *β_ii_*, and *β_ij_* are the regression coefficients for the intercept, linear, quadratic, and interaction terms, respectively, and *X_i_* and *X_j_* are the independent variables [23]. The equations were examined by an ANOVA to remove the terms that were not significant (*p* > 0.05). P_lack-of-fit_, the determination coefficient (R^2^), and the adjusted R^2^ were used to evaluate the adequacy and quality of the fitting. The extract optimization was obtained using the desirability function [24] to maximize the phenolic and antioxidant activity extraction. An external validation was conducted in the optimized conditions to verify the prediction power of the models by comparing the predicted and experimental data. All the statistical analyses were performed using Statistica v. 13.5 software (TIBCO Software Inc., Palo Alto, CA, USA).

## 3. Results and Discussion

### 3.1. Optimization of Extraction of Phenolic Compounds from Araucaria angustifolia Bracts

The data regarding the total phenols, total flavonoids, condensed tannins levels, and antioxidant activity by the DPPH and ABTS of the phenolic-rich extracts from the *Araucaria angustifolia* bracts are shown in Table 2. The total phenolic compounds ranged statistically (*p* < 0.001) from 3.57 to 9.48 mg GAE/g. These results are superior to those reported by Peralta et al. [25] for *Araucaria angustifolia* seeds (0.175 and 0.211 mg CTE/g for raw and cooked seeds, respectively). The total phenolic content for the aqueous extract of the sterile bracts, the extraction performed using distilled water under reflux at 100 °C for 15 min, determined by Souza et al. [5], was 15.86 mg GAE/g of the bracts. The flavonoid content in the extracts varied statistically (*p* < 0.001) from 2.10 to 6.57 mg CTE/g. Regarding the tannin content, a significant difference was observed (*p* < 0.001), with levels ranging from 2.17 to 10.56 mg CTE/g.

The antioxidant activity observed in the extract of the sterile bracts varied statistically (*p* < 0.001) for both methods, with the DPPH ranging from 15.57 to 68.69 μmol TE/g, and the ABTS ranging from 28.09 to 77.40 μmol TE/g. Freitas et al. [26] observed lower values of the antioxidant capacity using DPPH and ABTS in the phenolic extracts of the *pinhão* coats obtained by an extraction with pure ethanol (DPPH = 9.14 μmol TE/g; ABTS = 26.4 μmol TE/g) and 80% ethanol solution (*v*/*v*) (DPPH = 8.62 μmol TE/g; ABTS = 49.10 μmol TE/g). Dorneles and Noreña [27] evaluated the extraction of the bioactive compounds from the bracts of *Araucaria angustifolia* employing the aqueous microwave-assisted extraction method (MAE). The authors observed higher levels of antioxidant activity (DPPH = 467.79 μmol TE/g; ABTS = 427.28 μmol TE/g), possibly due to the extraction method, since the parameters used influence the phenolic profile and, consequently, the antioxidant activity of the samples. According to the authors, the polarity of the solvent used is a parameter to be considered, and when using the MAE method, water is the most suitable solvent, since the extraction process of the phenolic compounds is facilitated, resulting in higher concentrations in the samples.

The mathematical models built for each response variable by the multiple regression method demonstrated a significance at the 95% confidence level (*p* < 0.01) without a lack of fit (*p* > 0.05). These models showed a good fit with the experimental data (R^2^ > 0.89), and the adjusted R^2^ values were able to explain 84% to 98% of the data variation (Table 3).

The solvent concentration showed positive linear regression coefficients, indicating that this parameter had a positive influence on the extraction of the phenolic compounds and antioxidant activity, mainly for tannins. However, it showed a negative quadratic response for the total phenols, indicating that the intermediate concentration resulted in a higher extraction yield. The concentration of the solvent is directly related to the affinity of the compounds with the solvent, in other words, an extract obtained with a low concentration of ethanol contains a higher content of hydrophilic compounds, whereas when using a high concentration of ethanol, the extract will be rich in lipophilic compounds [28,29]. Many studies have reported that the use of a binary solvent system results in a greater extraction of phenolic compounds compared to the mono-solvent system (water or pure ethanol). A mixture between solvent and water has a greater effect on the extraction than pure solvent itself because when water is added to the solvent, it does not become so selective, therefore extracting more compounds [10,29,30].

Regarding the solute/solvent ratio, the linear regression coefficients showed that this parameter had a positive influence on the extraction of the phenolic compounds from the sample, while the quadratic regression coefficients showed a significant response with a negative effect. The higher the solvent/solid ratio, the greater the extraction yield, according to the principles of a mass transfer, which include the driving force during the mass transfer and the concentration gradient between the solid and the solvent, which is greater when a higher solvent/solid ratio is used [11]. According to the model, the highest yield would be obtained using a solute/solvent ratio of 1:40 (*w*/*v*).

Concerning the temperature, the linear regression coefficients had a significant influence, with a positive effect. Higher temperatures increase the solubility of the phenolic compounds because the increase in temperature facilitates the separation of polyphenols by breaking down the cellular constituents of the plant matrix, increasing the permeability of the cell membrane and facilitating the penetration of the solvent [31]. The highest yields were observed using a temperature of 80 °C. This temperature was suitable because the antioxidant activity was not significantly altered in the tests at 80 °C, showing that these compounds can resist this temperature without losing their characteristics.

The total phenolic compounds, flavonoids, and tannins showed a significant correlation (*p* ≤ 0.05) with the antioxidant activity by the DPPH method (r = 0.95; r = 0.97; and r = 0.96, respectively) and by the ABTS method (r = 0.97; r = 0.97; and r = 0.91, respectively). Previous studies have demonstrated a positive correlation between the phenolic compounds and antioxidant activity, which can be explained by the redox capacity of these compounds [5,32].

The optimization procedure was conducted to maximize the extraction of the total phenolic content, total flavonoids, tannins, and antioxidant activity by DPPH and ABTS. The optimization suggested an extraction with 60% ethanol and a ratio of 1:38 (*w*/*v*) at 80 °C. The new extraction procedures were performed at the optimal conditions to externally validate the model. The observed and predicted values, along with the computed absolute errors (AE) for the extraction, were: the total phenolics (mg/g) (observed: 9.75 ± 0.37; predicted: 10.10; AE = 3.57%); total flavonoids (mg/g) (observed: 6.73 ± 0.29; predicted: 6.77; AE = 0.56%); tannins (mg/g) (observed: 12.56 ± 0.53; predicted: 11.06; AE = 12.00%); ABTS (μmol TE/g) (observed: 84.07 ± 4.31; predicted: 81.92; AE = 2.62%); and DPPH (μmol TE/g) (observed: 68.53 ± 5.41; predicted: 66.63; AE = 2.84%).

### 3.2. The Individual Phenolic Compounds at the Optimized Point

The phenolic-rich extract from the *Araucaria angustifolia* bracts obtained at the optimized point (Section 3.1) were quantified as having 158.38 ± 1.02 µg/g of gallic acid, 353.44 ± 2.25 µg/g of catechin, 204.65 ± 2.29 µg/g of epicatechin, 154.04 ± 1.14 µg/g of quercetin, and 81.68 ± 0.37 µg/g of kaempferol. Souza et al. [5] performed an extraction using distilled water under reflux at 100 °C for 15 min and the reported contents were 1406 ± 28.6 µg/g of catechin, 413 ± 27.3 µg/g of epicatechin, and 232 ± 0.6 µg/g of quercetin from the *Araucaria angustifolia* bracts. These figures were higher than those of the present study, possibly due to the extraction method. In a study of *Araucaria angustifolia* by Michelon et al. [4], the authors observed levels of catechin (2810 ± 130 µg/g sterile bracts), epicatechin (740 ± 40 µg/g sterile bracts), and rutin (260 ± 30 µg/g sterile bracts). Gallic acid, catechin, and quercetin were also found in seeds (*pinhão*), and epicatechin was detected in the pine bark [1].

The phenolic compounds, which were also observed in the extract obtained from the sterile bracts, are found in different plant matrices and are widely studied due to their antioxidant capacity. Gallic acid can be found in several plant sources, such as the tea, grapes, and leaves of *Ficus auriculata* and *Moringa oleifera* [33,34,35], and its antioxidant capacity is related to the phenolic acids. Catechin and epicatechin are flavanols that are often found in fruits, vegetables, and tea [36]. Quercetin and kaempferol are flavonols that are particularly present in black and green tea, as well as fruits such as pomegranate and banana [37,38]. Both flavanols and flavonols have an antioxidant capacity associated with flavonoids, which causes a high redox potential, and therefore, a high reducing capacity (antioxidant action).

### 3.3. Antimicrobial Activity of the Optimized Extract

The minimum inhibitory concentration is the ability of an extract to inhibit a microorganism by 50% or 90%. The concentrations that presented antimicrobial activity against pathogenic and acid lactic strains are shown in Table 4. A concentration of 10,000 µg/mL produced the best inhibitions of *S. aureus*, *Enterococcus* spp., *Salmonella* spp., and *E. coli*. At a concentration of 5000 µg/mL, the best inhibition was in relation to *Enterococcus* spp., *Salmonella* spp., and *E. coli*. At concentrations of 2500, 1250, and 625 µg/mL, the best inhibition was for *Enterococcus* spp. However, at this concentration, the application of the extract can be complex, arising from a possible toxicity and changes that may occur in the final product. Therefore, future studies should focus on the application of this extract in packaging and as an insecticidal agent. At the other concentrations, the inhibition was most prominent in relation to *Enterococcus* spp. The bacteria of the genus *Enterococcus* are of great importance in the area of food as they work as the indicators for the contamination of the fecal origin; this bacteria is present in the intestinal tract of homoeothermic mammals and is very resistant [39].

The lower level of inhibition observed for the *Lactobacillus brevis* strain (approximately 28% of the inhibition at the highest analyzed concentration) may have been due to the increased resistance. Because this bacterium has adapted to hostile growth environments, such as gaining a high acidity, the presence of ethanol, and the components with antimicrobial action derived from hops, *Lactobacillus brevis* is used in sour beer brewing [40]. The extracts from *Araucaria angustifolia* seed residues demonstrated an inhibitory growth capacity against pathogenic strains (*Staphylococcus aureus*, *Bacillus cereus*, *Listeria monocytogenes*, *L. innocua*, and *Aeromonas hydrophila*) [41]. Therefore, the extracts from this plant matrix have antimicrobial activity against pathogenic bacteria and may represent an alternative barrier mechanism for food preservation.

### 3.4. Antiglycemic Activity

The antiglycemic activity (Table 5) was evaluated for the inhibitory activity of α-glucosidase. Values of 0.58 mg/mL were observed for the phenolic extract, which was ten times higher than the control of acarbose (IC_50_ = 5.54 mg/mL), which is a drug used as an α-glucosidase inhibitor. Therefore, the phenolic extract from the sterile bracts of *Araucaria angustifolia* can be explored as an alternative antiglycemic agent. There is a demand for new agents with antiglycemic action because the drugs which are currently used for this purpose have many side effects, such as acute hypoglycemia, lipodystrophy, metallic taste, lactic acidosis, vitamin B12 deficiency, weight gain, and gastrointestinal problems [42].

According to Lu et al. [43], phenolic acids (p-hydroxybenzoic, protocatechuic, p-coumaric, and caffeic) and flavonoids (quercetin, catechin, and epicatechin) were mainly detected in the seed coat of immature fava beans (*Vicia faba* L.), demonstrating that the antiglycemic activity that was observed (IC_50_ between 0.05 and 0.14 mg/mL) was possibly due to the phenolic profile of the extract of the analyzed plant matrix. Schmeda-Hirschmann et al. [44] observed that the high inhibitory activity of α-glucosidase for the phenolic extracts of boiled Chilean *Araucaria araucana* grains were possibly related to the high phenolic content of the husks. Furthermore, the phenolic compounds were solubilized in the water used as a solvent for the extraction, since the phenolic extract obtained from the decoction water showed a high enzyme inhibitory activity (IC_50_ ranging from 0.21 to 0.94 μg/mL), which was superior to the values of the α-glucosidase inhibitory activity of the phenolic extract of the sterile bracts of *Araucaria angustifolia*.

### 3.5. Cellular Antioxidant and Anti-Inflammatory Activity

The cellular antioxidant activity (CAA) of the phenolic extract of the sterile bracts of *Araucaria angustifolia* was evaluated (Table 6) and no antioxidant activity was observed, even at the highest tested concentration (2000 µg/mL). Comparatively, the quercetin control showed an inhibition of 95 ± 5% at a concentration of 0.3 µg/mL. Wolfe and Liu [20] evaluated the cellular antioxidant activity of the fruit extracts, and the extraction was performed using 80% acetone. The authors found that the greatest efficiency was provided by blueberry (EC_50_ = 3.44 mg/mL) in inhibiting the peroxyl radical-induced DCFH (2’,7’-dichlorofluorescin) oxidation, followed by cranberry (EC_50_ = 11.31 mg/mL), apple (EC_50_ = 21.31 mg/mL), red grape (EC_50_ = 24.49 mg/mL), and green grape (EC_50_ = 62.89 mg/mL). Nepali hog plum (*Choerospondias axillaris*) bark showed strong cellular antioxidant activity in relation to Caco-2 cells (EC_50_ = 10.2 ± 1.4 and 38.9 ± 2.1 μg/mL without or with phosphate-buffered saline washing, respectively), thus, these extracts can reduce oxidative stress in cells [45].

In the present study, the extract did not exhibit anti-inflammatory activity (Table 6), even at the highest tested concentration (400 μg/mL), whereas the dexamethasone control presented an IC_50_ of 6.3 ± 0.4 μg/mL. A similar result was observed by Oliveira et al. [46] when analyzing the extracts from the residues of *pinhão* (*Araucaria angustifolia* (Bertol.) Kuntze) seeds; the authors did not verify any anti-inflammatory activity in the tested concentration range (up to 400 μg/mL).

Non-steroidal anti-inflammatory drugs (NSAIDs) are one of the classes of drugs frequently used to treat inflammation. Like other medications, NSAIDs can cause adverse effects in the gastrointestinal system, mainly due to a prolonged use [47]. Thus, studies have investigated natural sources with an anti-inflammatory action that are possibly related to the phenolic profile of the samples. According to Talaat et al. [48], *Araucaria bidwillii* Hook. leaves have been shown to be an anti-inflammatory agent with a high reduction in inflammation, and consequently in its related risks. The extracts obtained from jabuticaba epicarp were related to the anti-inflammatory activity in relation to chronic obstructive pulmonary disease [49] and inhibited the growth of raw mouse macrophages 264.7 [50], possibly due to the presence of the methyl compound 2-[(3,4-dihydroxybenzoyloxy)-4,6-dihydroxyphenyl] acetate, which is mainly found in aboticaba bark. Although the mechanism of action is not yet fully elucidated, it is believed that the chemical structure of the phenolic compounds is directly related to the anti-inflammatory capacity and has a similar action to NSAIDs [47].

### 3.6. Antiproliferative Activity and Hepatotoxicity

Cancer is one of the diseases that causes most deaths worldwide. Treating cancer is very difficult, is debilitating, and reduces the quality of life of the individuals affected by the disease. The administration of drugs with genotoxic and cytotoxic actions is one of the treatments applied in patients affected by cancer. Such drugs act on cell proliferation and can result in cytotoxicity in many types of cells that are in the process of cell division, thus, they can have side effects such as nausea, gastrointestinal disturbance (diarrhea and changes in the gut microbiota), hair loss, fatigue, and immunosuppression [51,52]. Foods that are rich in phenolic compounds have been presented as an alternative to help prevent the development of abnormal cells. The phenolic extract of the sterile bracts of *Araucaria angustifolia* was evaluated for its inhibiting activity in the growth of three tumor cell lines (Table 7).

The extract was able to inhibit the development of tumor cells, with GI_50_ values ranging from 55 to 171 μg/mL for stomach and breast carcinoma, respectively, and these values were higher than those observed for the ellipticine control, which reflects the fact that the control had a higher antiproliferative potential. However, the extract of the sterile bracts showed cytotoxic activity for the analyzed non-tumor cells (PLP2 and VERO) at lower GI_50_ values, except for when the VERO cell line was compared to the stomach carcinoma cell line. Thus, a lower concentration is necessary to express the inhibition of cell proliferation in non-tumor cells, demonstrating the toxicity of the extract of sterile bracts of *Araucaria angustifolia* to normal cells.

Anticancer drugs are designed to be effective in the treatment of malignant cancer disorders; however, they can have toxic effects on normal cells [50]. Thus, a cytotoxic selectivity index (CSI) (Table 8) was verified for the extract of sterile bracts of *Araucaria angustifolia*, and the ellipticine control was used for a comparison. The ellipticine control did not show a selectivity for the tumor cells, with an average CSI of 1.2, considering the cell line of the analyzed tumoral and normal cells. Similar behavior was observed for the phenolic extract of *Araucaria angustifolia* (Table 8). Ellipticine presents as an anticancer mechanism of action in terms of a DNA intercalation, the inhibition of DNA topoisomerase II activity, and covalent binding to DNA, inducing cell cycle arrest and the induction of apoptosis [53]. Thus, considering that the target of antitumor drugs, such as ellipticine, is cell division, such drugs may possibly affect, to a lesser or greater degree, all the normal tissues undergoing a cell division. Girardelo et al. [54] observed a low selectivity index of gemcitabine, a gold standard drug for the treatment of pancreatic cancer, due to low CC_50_ values for the non-tumor cells HUVEC and VERO (human umbilical vein endothelial, and monkey kidney epithelial cell lines, respectively).

Considering the possible toxic effects of antitumor drugs on normal cells, alternative natural sources have been investigated. Oliveira et al. [46] evaluated the bioactive properties of pine nut seed residue extracts obtained by cooking in water and the ethanol extract from the shells and observed that the aqueous extract did not present an inhibition capacity for any of the cancer cells investigated. However, the ethanol extract showed cytotoxic activity for all the tumor lines and was most effective against hepatocellular carcinoma. In addition, even at the highest concentration evaluated (400 μg/mL), both extracts did not present a cytotoxic effect in normal porcine liver cells (without carcinoma), indicating that the extract did not affect non-tumor cells. This difference in toxicity in relation to the present study may be related to the phenolic content, possibly due to the extraction method employed, as well as the intrinsic factors regarding the samples.

Plants present bioactive compounds in their composition and, consequently, they may present a toxic potential due to their chemical constituents. Therefore, some plants used as food and/or in traditional medicine have been related to toxic effects, such as the mutagenic and carcinogenic activity. Puncture vine (*Tribulus terrestris*) has been used as a performance-enhancing supplement for rapid gains in strength and muscle mass. However, cases of renal toxic effects (secondary nephrotoxicity), a decreased or interrupted bile flow (cholestasis), and hyperbilirubinemia have been reported according to Ryan et al. [55].

In many cases, the toxic effect observed in the plant extracts is dose-dependent, and a combination of the plants, which alone do not present toxic effects, can cause harmful effects, as observed by Ala et al. [56]. The aforementioned authors observed a cytotoxic effect of the combination of the extracts of the two plants combined in a 1:1 ratio, with a reduction in the cellular viability of spleleocytes (89% to 35%), thynocytes (67% to 28%), and hepatocytes (69% to 64%); this reduction in cell viability was linked to increased concentrations (4 μg/mL–500 μg/mL). In other words, the incubation of the combined extracts for 72 h was toxic to the cells, and the level of cell damage was concentration-dependent. A test performed by Kapetanovic et al. [57] demonstrated that the incidence of mortality in a nine-month chronic canine toxicology study of highly purified and standardized green tea extract (Polyphenon E^®^) was dose-dependent. Ewing et al. [58] observed that the administration of a concentrated extract of cannabis enriched with cannabidiol in mice showed hepatotoxic effects at a concentration of 2.46 mg/kg, demonstrating the potential of the extract to cause liver damage in mice.

Therefore, administering the correct dose of medicinal plants and foods is essential to provide the benefits that bioactive compounds can provide, without adverse reactions or harmful effects on the cells [59]. Further studies should be conducted to elucidate information on the bioavailability and bioactivity of the foods added to the phenolic extracts of the sterile bracts of *Araucaria angustifolia*, and thus obtain more information on the toxicity of the extract in normal cells.

## 4. Conclusions

The phenolic compounds present in sterile bracts can be obtained using ethanol as a solvent from optimized parameters (60% ethanol, at 80 °C with a 1:38 solute/solvent ratio (*w*/*v*)). The response surface methodology was efficient in predicting the effects of the independent variables (solvent concentration, solute/solvent ratio, and extraction temperature) in the extraction of the total phenolic compounds, total flavonoids, tannins, and antioxidant activity by the DPPH and ABTS methods. The extract showed antimicrobial activity for the analyzed pathogenic and lactic bacteria and, at the lowest investigated concentrations, lactic acid bacteria had the greatest resistance. The extract showed a high inhibition of the α-glucosidase enzyme, indicating an excellent antiglycemic potential and high antiproliferative properties against tumor cells. However, the extract demonstrated toxicity in relation to PLP2 and VERO cells and did not show a cellular antioxidant and anti-inflammatory activity, which it is in agreement with the literature regarding this plant matrix. Therefore, the phenolic extract of the sterile bracts of *Araucaria angustifolia* has a high bioactive potential and can contribute to the fight against degenerative diseases, which can be explored in the development of new products, provided that safe concentrations related to bioavailability assays are investigated.

## Figures and Tables

**Table 1:** Box–Behnken design for phenolic extraction of bracts from *Araucaria angustifolia*.

Assay	Factors		
	Concentration (%)	Ratio (*w*/*v*)	Temperature (°C)
1	20 (−1)	10 (−1)	55 (0)
2	60 (+1)	10 (−1)	55 (0)
3	20 (−1)	40 (+1)	55 (0)
4	60 (+1)	40 (+1)	55 (0)
5	20 (−1)	25 (0)	30 (−1)
6	60 (+1)	25 (0)	30 (−1)
7	20 (−1)	25 (0)	80 (+1)
8	60 (+1)	25 (0)	80 (+1)
9	40 (0)	10 (−1)	30 (−1)
10	40 (0)	40 (+1)	30 (−1)
11	40 (0)	10 (−1)	80 (+1)
12	40 (0)	40 (+1)	80 (+1)
13	40 (0)	25 (0)	55 (0)
14	40 (0)	25 (0)	55 (0)
15	40 (0)	25 (0)	55 (0)

**Table 2:** Total phenolic compounds (TPC), total flavonoids (TF), tannins (TAN), and antioxidant capacity by DPPH and ABTS from *Araucaria angustifolia* bracts.

Assay	TPC (mg GAE/g)	TF (mg CTE/g)	TAN (mg CTE/g)	DPPH (μmol TE/g)	ABTS (μmol TE/g)
1	3.57 ^h^ ± 0.12	2.10 ^l^ ± 0.05	2.17 ^n^ ± 0.11	22.30 ^g^ ± 1.47	28.09 ^i^ ± 1.21
2	4.64 ^g^ ± 0.28	3.14 ^ij^ ± 0.15	4.83 ^i^ ± 0.19	23.30 ^g^ ± 0.85	33.44 ^h^ ± 0.85
3	6.89 ^e^ ± 0.44	4.29 ^h^ ± 0.08	4.03 ^j^ ± 0.13	31.48 ^f^ ± 3.05	52.34 ^ef^ ± 1.60
4	8.66 ^b^ ± 0.42	5.68 ^c^ ± 0.10	9.40 ^c^ ± 0.38	54.89 ^c^ ± 2.60	73.32 ^b^ ± 1.62
5	4.92 ^g^ ± 0.20	3.03 ^j^ ± 0.08	2.97 ^l^ ± 0.13	22.20 ^g^ ± 3.16	32.58 ^h^ ± 0.67
6	7.46 ^cd^ ± 0.37	4.88 ^def^ ± 0.07	7.44 ^d^ ± 0.32	45.88 ^d^ ± 1.08	50.65 ^f^ ± 1.80
7	7.86 ^c^ ± 0.12	4.81 ^efg^ ± 0.16	6.95 ^e^ ± 0.28	45.64 ^d^ ± 1.41	53.08 ^e^ ± 0.49
8	9.27 ^a^ ± 0.31	6.05 ^b^ ± 0.18	10.56 ^a^ ± 0.30	60.55 ^b^ ± 1.34	74.22 ^b^ ± 2.03
9	3.77 ^h^ ± 0.15	2.12 ^l^ ± 0.09	2.60 ^m^ ± 0.13	15.57 ^h^ ± 1.15	28.13 ^i^ ± 0.77
10	7.24 ^de^ ± 0.22	4.57 ^g^ ± 0.10	5.23 ^gh^ ± 0.23	37.97 ^e^ ± 1.18	53.51 ^e^ ± 2.03
11	5.76 ^f^ ± 0.27	3.36 ^i^ ± 0.05	5.00 ^hi^ ± 0.09	29.55 ^f^ ± 0.98	41.13 ^g^ ± 1.28
12	9.48 ^a^ ± 0.34	6.57 ^a^ ± 0.25	10.26 ^b^ ± 0.22	68.69 ^a^ ± 3.56	77.40 ^a^ ± 2.05
13	7.59 ^cd^ ± 0.22	4.94 ^de^ ± 0.14	6.18 ^f^ ± 0.14	43.12 ^d^ ± 1.98	50.49 ^f^ ± 2.16
14	7.37 ^d^ ± 0.16	4.66 ^fg^ ± 0.21	5.51 ^g^ ± 0.19	38.80 ^e^ ± 2.56	57.30 ^d^ ± 0.41
15	7.81 ^c^ ± 0.37	5.09 ^d^ ± 0.28	6.39 ^f^ ± 0.21	44.22 ^d^ ± 1.55	64.11 ^c^ ± 1.78
*p* (Hartley) *	0.70	0.49	0.08	0.31	0.49
*p* (one-way ANOVA) **	<0.001	<0.001	<0.001	<0.001	<0.001

GAE: gallic acid equivalent; CTE: catechin equivalent; TE: Trolox equivalent; DPPH: 2,2-diphenyl-1-picrylhydrazyl assay; ABTS: 2,2′-azino-bis(3-ethylbenzothiazoline-6-sulfonic acid) radical cation-based assay; * probability values obtained by Hartley test for homogeneity of variances; ** probability values obtained by one-way ANOVA. Different letters in the same column represent statistical differences according to the Fischer LSD test (*p* ≤ 0.05).

**Table 3:** Effects of independent variables (solvent concentration, solute/solvent ratio, and temperature) for the evaluation of dependent variables using response surface methodology.

Response Variable	Factors	Regression Coefficient	Standard Error	t-Value	*p*-Value	−95% Confidence	+95% Confidence
Total phenolic compounds (mg/g)	Constant	7.71	0.15	50.85	<0.001	7.37	8.06
	$x_{1}^{}$	0.85	0.11	7.59	<0.001	0.59	1.10
	$x_{1}^{2}$	−0.43	0.16	−2.63	0.03	−0.80	−0.06
	$x_{2}^{}$	1.81	0.11	16.24	<0.001	1.56	2.07
	$x_{2}^{2}$	−1.25	0.16	−7.61	<0.001	−1.62	−088
	$x_{3}^{}$	1.12	0.11	10.06	<0.001	0.87	1.38
	R^2^	0.98					
	Adjusted R^2^	0.97					
	*p*-value (model)	<0.001					
	*p*-value (lack of fit)	0.33					
Total flavonoids (mg/g)	Constant	4.78	0.11	43.35	<0.001	4.53	5.02
	$x_{1}^{}$	0.69	0.10	6.70	<0.001	0.46	0.92
	$x_{2}^{}$	1.30	0.10	12.60	<0.001	1.07	1.53
	$x_{2}^{2}$	−0.80	0.15	−5.31	<0.001	−1.13	−0.46
	$x_{3}^{}$	0.77	0.10	7.49	<0.001	0.54	1.00
	R^2^	0.97					
	Adjusted R^2^	0.95					
	*p*-value (model)	<0.001					
	*p*-value (lack of fit)	0.38					
Tannins (mg/g)	Constant	6.55	0.31	20.93	<0.001	5.85	7.24
	$x_{1}^{}$	2.00	0.29	6.82	<0.001	1.34	2.65
	$x_{2}^{}$	1.79	0.29	6.11	<0.001	1.14	2.44
	$x_{2}^{2}$	−1.11	0.43	−2.59	0.03	−2.06	−0.15
	$x_{3}^{}$	1.79	0.29	6.13	<0.001	1.14	2.45
	R^2^	0.93					
	Adjusted R^2^	0.90					
	*p*-value (model)	<0.001					
	*p*-value (lack of fit)	0.22					
ABTS (μmol TE/g)	Constant	54.63	1.97	27.71	<0.001	50.24	59.03
	$x_{1}^{}$	8.19	1.84	4.44	<0.001	4.08	12.30
	$x_{2}^{}$	15.72	1.84	8.52	<0.001	11.61	19.83
	$x_{2}^{2}$	−6.21	2.70	−2.30	0.04	−12.23	−0.20
	$x_{3}^{}$	10.11	1.84	5.49	<0.001	6.01	14.23
	R^2^	0.93					
	Adjusted R^2^	0.90					
	*p*-value (model)	<0.001					
	*p*-value (lack of fit)	0.81					
DPPH (μmol TE/g)	Constant	42.91	2.29	18.70	<0.001	37.80	48.03
	$x_{1}^{}$	7.87	2.15	3.67	<0.01	3.09	12.66
	$x_{2}^{}$	12.79	2.15	5.96	<0.001	8.00	17.57
	$x_{2}^{2}$	−7.44	3.14	−2.37	0.04	−14.44	−0.44
	$x_{3}^{}$	10.35	2.15	4.82	<0.001	5.57	15.13
	R^2^	0.89					
	Adjusted R^2^	0.84					
	*p*-value (model)	<0.001					
	*p*-value (lack of fit)	0.17					

*x*_1_: ethanol concentration: *x*_2_: solute-solvent ratio; *x*_3_: temperature; TE: Trolox equivalent; DPPH: 2,2-diphenyl-1-picrylhydrazyl assay; ABTS: 2,2′-azino-bis(3-ethylbenzothiazoline-6-sulfonic acid) radical cation-based assay.

**Table 4:** Antimicrobial activity of extract of sterile bracts of *Araucaria angustifolia*.

Strain	Concentration (μg/mL)	Inhibition (%)
*Staphylococcus aureus*	10,000	81.3
*Enterococcus* spp.	10,000	86.9
	5000	76.3
	2500	82.8
	1250	63.7
*Salmonella* spp.	10,000	83.5
	5000	59.4
*Escherichia coli*	10,000	79.7
	5000	57.5
*Lactobacillus brevis*	15,000	27.9
	10,000	19.5

**Table 5:** Antiglycemic activity of sterile bracts extract of *Araucaria angustifolia*.

Antiglycemic Activity	IC_50_ (mg/mL)
Phenolic extract	0.58
Acarbose control	5.54

**Table 6:** Cellular antioxidant and anti-inflammatory activity of extract of sterile bracts of *Araucaria angustifolia*.

Activity	Concentration (µg/mL)	Activity
Cellular antioxidant (Inhibition, %)		
Phenolic extract	2000	ND *
Quercetin control	0.3	95 ± 5%
Anti-inflammatory (IC_50_)		
Phenolic extract	400	ND *
Dexamethasone control	-	6.3 ± 0.4

* ND: not detected.

**Table 7:** Antiproliferative and cytotoxicity activity of extract of sterile bracts of *Araucaria angustifolia*.

Antiproliferative Activity GI_50_ (μg/mL)	Phenolic Extract	Ellipticine
AGS (gastric carcer cell line)	55 ± 5	1.23 ± 0.03
CaCo-2 (colon carcinoma cell line)	140 ± 7	1.21 ± 0.02
MCF-7 (breast cancer cell line)	171 ± 19	1.02 ± 0.02
PLP2 (non-tumor cell line)	41 ± 3	1.4 ± 0.1
VERO (non-tumor cell line)	75 ± 7	1.41 ± 0.06

**Table 8:** Cytotoxic selectivity index (CSI) of the phenolic extract of sterile bracts of *Araucaria angustifolia* and the ellipticine control.

	Cytotoxic Selectivity Index (CSI)					
	PLP2			VERO		
	AGS	CaCo2	MCF-7	AGS	CaCo2	MCF-7
Phenolic extract	0.75	0.29	0.24	1.36	0.54	0.44
Ellipticine	1.14	1.16	1.37	1.15	1.17	1.38

PLP2 and VERO: non-tumor cells; AGS: stomach carcinoma; CaCo2: bowel carcinoma; MCF-7: breast carcinoma.

## Data Availability

Not applicable.
